# Liver metastases from pituitary carcinomas mimicking visceral well-differentiated neuroendocrine tumors: a series of four cases

**DOI:** 10.1186/s13000-020-00997-x

**Published:** 2020-07-04

**Authors:** Elise R. Venable, Sarah E. Kerr, M. Beatriz S. Lopes, Karra A. Jones, Andrew M. Bellizzi, Taofic Mounajjed, Aditya Raghunathan, Oksana Hamidi, Thorvardur R. Halfdanarson, Mabel Ryder, Rondell P. Graham

**Affiliations:** 1 Division of Anatomic Pathology, Mayo Clinic, 200 First St SW, Rochester, MN 55905 USA; 2 Department of Pathology, University of Virginia Health System, Charlottesville, VA USA; 3 Department of Pathology, University of Iowa, Iowa City, IA USA; 4 Division of Endocrinology, Department of Internal Medicine, Mayo Clinic, Rochester, MN USA; 5 Division of Endocrinology and Metabolism, University of Texas Southwestern Medical Center, Dallas, TX USA; 6 Division of Medical Oncology, Department of Internal Medicine, Mayo Clinic, Rochester, MN USA

**Keywords:** Pituitary tumor, Pituitary carcinoma, Cushing syndrome, Neuroendocrine tumor, Liver metastasis

## Abstract

**Background:**

Pathologists frequently encounter neuroendocrine tumors (NETs) presenting as multiple liver masses in routine practice. Most often, these are well-differentiated tumors with characteristic histologic features. In contrast, pituitary carcinoma is very rare, and there is limited data on its natural history and pathologic characterization.

**Methods:**

The aim of this study was to describe clinical characteristics, histomorphology, immunophenotype and follow-up of pituitary carcinoma involving the liver and mimicking well-differentiated NETs of visceral origin. We selected a group of well-differentiated NETs of the pancreas to use as immunophenotypic controls. We identified 4 patients (age range, 51 to 73) with pituitary corticotroph carcinoma with liver metastases. Three patients presented with Cushing syndrome.

**Results:**

All cases histologically resembled well-differentiated NETs of visceral origin with Ki-67 proliferation indices of 5–42% and expression of T-PIT; metastatic tumors were not immunoreactive with CDX2, Islet 1 or TTF-1.

**Conclusions:**

Frequently, these cases display adrenocorticotropic hormone (ACTH) secretion and pituitary-specific transcription factor immunohistochemistry may be used as a reliable marker to distinguish metastatic pituitary carcinoma from NETs of visceral origin in addition to delineating a corticotroph carcinoma from somatotroph, lactotroph, thyrotroph, and gonadotroph lineage. Although rare, the differential diagnosis of pituitary carcinoma should be considered in metastatic well-differentiated NETs in which the site of origin is uncertain. In summary, pituitary corticotroph carcinoma can metastasize to the liver and mimic well-differentiated NET.

## Background

Pathologists frequently encounter neuroendocrine neoplasms presenting as multiple liver masses [1]. Despite being considered an uncommon disease [2], the incidence and prevalence of neuroendocrine tumors is growing in the United States and elsewhere around the globe [2–4]. More than 50% of neuroendocrine tumors within the body arise in the gastrointestinal (GI) tract and pancreas [5] and approximately one half of gastrointestinal cases have liver metastases at presentation [6, 7]. Bronchopulmonary neuroendocrine tumors are less common [7] but the lung and other sites may give rise to metastatic neuroendocrine tumors as well. Consequently, immunohistochemistry and less commonly molecular tests are used to identify the primary site [8–10]. In practice, when a metastatic neuroendocrine tumor is identified, the primary sites typically considered are the GI tract and pancreas followed by lung. These are typically well-differentiated tumors with characteristic cytomorphologic and/or histologic features. Less commonly, these are poorly differentiated neuroendocrine carcinomas, which differ in their morphology, molecular biology and differential diagnosis [11–13]. In our practices we have encountered rare cases of pituitary carcinoma metastatic to the liver simulating well-differentiated neuroendocrine tumors (NETs). Pituitary carcinomas deriving from the adenohypophysis are exceedingly rare accounting for 0.1–0.5% of all pituitary tumors with only limited reports of its natural history [14, 15]. Experts in the field of pituitary neoplasia have suggested the term pituitary neuroendocrine tumor (PitNET) rather than continuing the use of the term pituitary adenoma because of the risk of inappropriate terminology in cases with eventual metastases [16]. The World Health Organization (WHO) team endorsed this new nomenclature as part of an effort to harmonize the diagnostic terminology to neuroendocrine tumors at various sites [17]. The aim of this study was to describe the clinicopathologic features of a series of pituitary corticotroph carcinomas involving the liver and mimicking well-differentiated neuroendocrine tumors of visceral origin.

## Materials and methods

### Cases

This study was approved by the Mayo Clinic Institutional Review Board. The authors (SEK, KAJ, AMB and RPG) encountered 2 cases of pituitary carcinoma in liver cytology/biopsy specimens. We attempted a search of our pathology databases and identified another 2 cases over a 21 year period (January 1997 to April 2018). The archived diagnostic slides of these cases were retrieved. A representative formalin fixed paraffin tissue block was selected for ancillary immunohistochemistry. The patients’ medical records were reviewed for clinical information including dates of diagnosis, radiologic findings, serum hormone levels, clinical presentation and follow-up.

### Controls

In our index case, the patient was clinically thought to have hepatic metastases from pancreatic NET due to the presence of a pancreatic mass. Therefore, four (4) cases of well-differentiated NETs of the pancreas were randomly selected from the pathology database as controls for immunohistochemistry. We also included a fifth control case of pancreatic NET characterized by ACTH secretion and ectopic Cushing syndrome.

### Immunohistochemistry

Immunohistochemistry was performed on formalin fixed tissue sections using the following antibodies at Mayo Clinic: OSCAR cytokeratin (clone OSCAR, predilute, BioLegend, Dedham, MA), chromogranin A (clone LK2H10, predilute, Ventana, AZ), CDX2 (clone EPR2764Y, 1/200, Cell Marque, Rocklin, CA), Islet 1 (clone 1H9, 1/800, abcam, Cambridge, MA), INSM1 (clone A8, 1/100, Santa Cruz, CA), Ki-67 (clone MIB-1, 1/20, Dako, Carpinteria, CA) and TTF-1 (clone SPT24, 1/100, Leica, Newcastle, UK). INSM1 and chromogranin were used as neuroendocrine markers. First, the sections were deparaffinized then rehydrated and stained online using antibody specific epitope retrieval techniques with the Ventana Benchmark XT system (Ventana, AZ).

Immunohistochemistry was performed at the University of Virginia Health System for T-PIT using the TBX19 antibody (clone T-PIT, 1/2000, Atlas Antibodies AB, Sweden) on the Ventana Benchmark platform. Immunohistochemistry for all markers was scored as follows: Negative (−) = 0% of cells staining and Positive (+) = > 10% of cells staining as an arbitrary minimum value. Automated Ki-67 analysis was performed using the digital method previously published by Kroneman et al. [18].

## Results

We identified 4 cases of pituitary carcinoma with liver metastases between January 1st, 1997 and April 30th, 2018. Of these, 2 cases were identified in a single year and the initial clinical concern was for involvement by well-differentiated NET of gastroenteropancreatic or visceral origin. The four patients (3 women and 1 man) were diagnosed with ACTH-secreting pituitary carcinoma with liver metastases at ages ranging from 51 to 73 years. Three patients presented with Cushing syndrome characterized by markedly elevated corticotropin (ACTH) levels at the time of diagnosis of the liver lesions (78,336, 33,000, 1056; normal range: 10–50 pg/ml) and had prior histories of ACTH-secreting pituitary tumors (19, 72, and 52 months prior to developing liver metastases, respectively). None of the patients had multiple endocrine neoplasia syndrome. Table 1 shows the clinical characteristics of the patients. All patients had multiple liver masses on abdominal computed tomography (CT) and magnetic resonance imaging (MRI). One patient (Patient #3) had a 2.5 cm pancreatic head mass concomitantly noted on the imaging. Upon comparative imaging review 1 year prior, the pancreatic mass previously measured 5.0 cm indicating that the pancreatic lesion had decreased in size. The patient underwent a biopsy of the pancreatic lesion, but it was non-diagnostic and showed normal pancreatic acini and fibrosis. None of the remaining patients had thoracic, pancreatic or other extrahepatic abdominal masses or intra-abdominal adenopathy detected by imaging studies.

**Table 1:** Summary of the clinical characteristics of the patients with pituitary carcinomas presenting as multiple liver masses

Name	Age at time of liver diagnosis	Interval between pituitary diagnosis and liver diagnosis (months)	Sex	Abdominal imaging findings	8 am Serum ACTH (pg/ml)	Status at follow up	Duration of follow up (months)
1	51	16	F	Multiple liver masses	78,336	Alive with disease	24
2	65	72	F	Multiple liver masses	33,000	Died of disease	86
3	73	52	M	Multiple liver masses	1056	Alive with disease	60
4	50	36	F	Multiple liver masses	unknown	Alive with disease	36

**Fig. 1:**
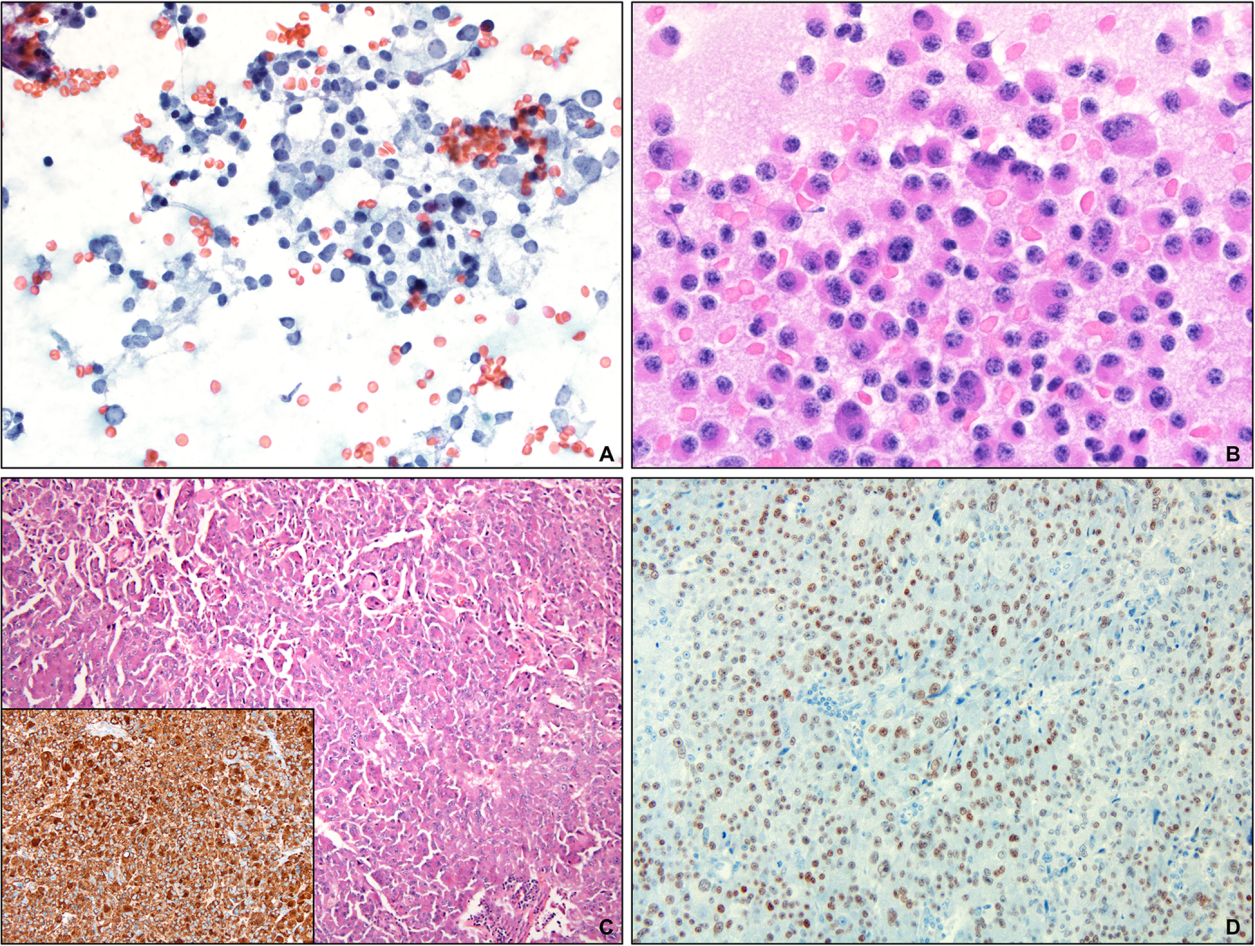
**a** Pap-stained cytologic smears from fine needle aspiration of the liver masses (Original magnification × 400) showing characteristic nuclear features of neuroendocrine neoplasm. **b** Diff Quik stained slides showing cells with eccentric nuclei and binucleate cells (Original magnification × 600). **c** The liver was infiltrated by monotonous cells with abundant eosinophilic cytoplasm, open chromatin and visible nucleoli (Original magnification × 100). Inset: The tumor cells were diffusely positive for chromogranin (Original magnification × 200). **d** The tumor cells were also diffusely positive for T-PIT immunohistochemistry (Original magnification × 200)

Two cases (Patients #2 and #3) were examined by fine needle aspiration and cytology preparations revealed discohesive populations of intermediate-size tumor cells with a modest amount of cytoplasm bearing nuclei with coarse salt and pepper type chromatin (Fig. 1). The nuclei were frequently eccentrically placed and occasional cells were binucleate (Fig. 1b).

**Fig. 2:**
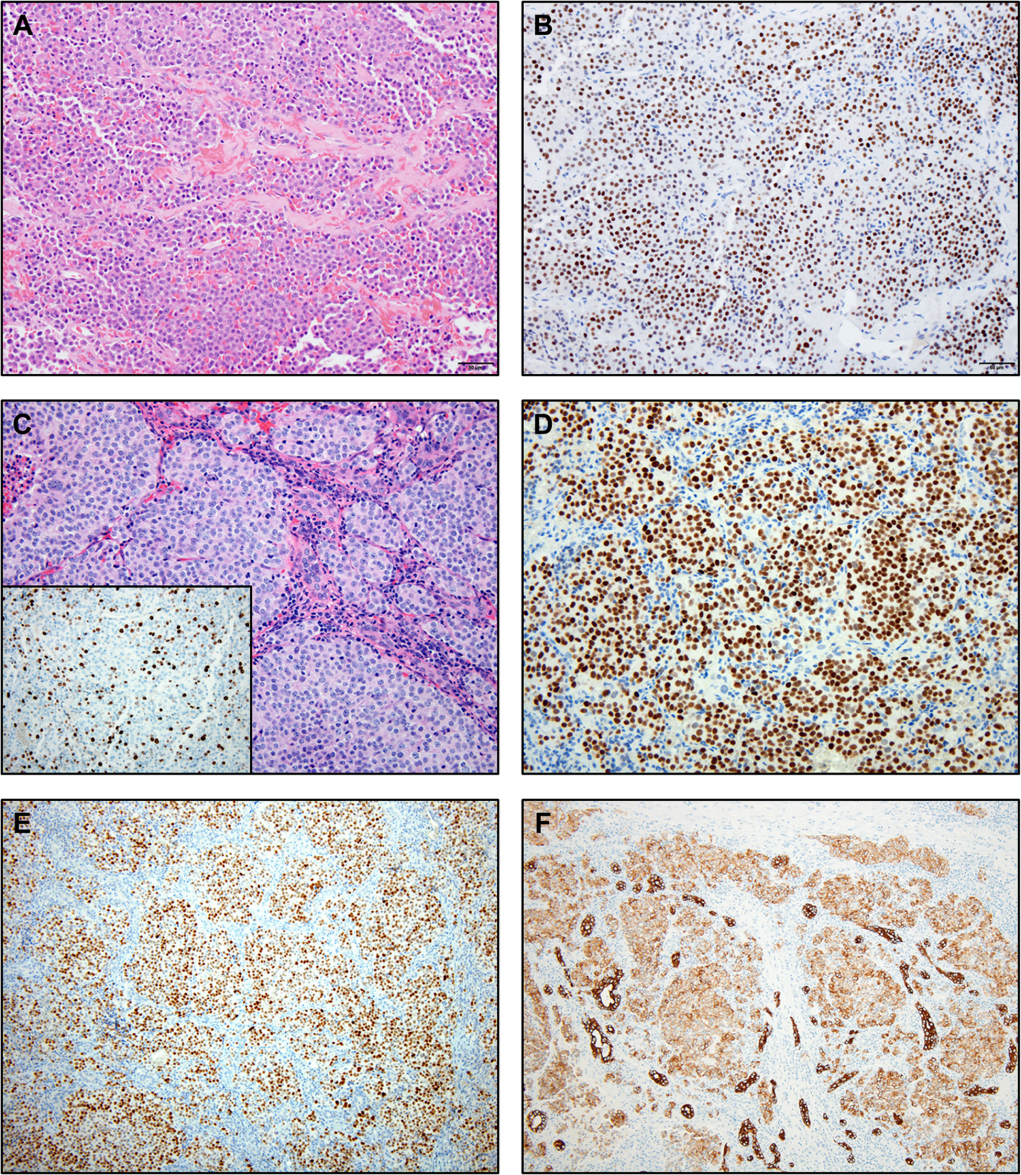
**a** This photomicrograph of the previously resected pituitary lesion shows monotonous neoplastic cells (Original magnification × 200). **b** The neoplastic cells show diffuse expression of the nuclear neuroendocrine marker insulinoma associated protein (INSM1) (Original magnification × 200). **c** The liver shows nodules of tumor cells with moderate amounts of cytoplasm, clumped chromatin and variably prominent nucleoli (Original magnification × 200). Inset: Ki-67 shows an elevated proliferative rate of more than 10% (Original magnification × 200). **d** The neuroendocrine marker, INSM1 was diffusely positive in the tumor cells (original magnification × 200). **e** The transcription factor, T-PIT is diffusely positive confirming pituitary origin of the tumor cells (Original magnification × 100). **f** OSCAR cytokeratin also highlighted the tumor cells (Original magnification × 100)

Histologically, all four cases showed similar findings (Fig. 2). The liver was infiltrated by a proliferation of monotonous neoplastic cells characterized by coarse chromatin and modest amounts of pale cytoplasm. These histologic and cytologic findings were suggestive of well-differentiated NET. Marked nuclear enlargement, nuclear irregularity, hyperchromasia, macronucleoli and atypical mitotic figures were not seen. None of the cases resembled small cell carcinoma or large cell neuroendocrine carcinoma.

By immunohistochemistry, all liver metastases from pituitary carcinoma were positive for T-PIT, OSCAR and the neuroendocrine markers chromogranin and INSM1. CDX2, TTF-1 and Islet1 were negative in all cases. ACTH immunohistochemical staining was positive in the single pituitary carcinoma case in which it was tested. Automated Ki-67 analysis highlighted proliferation indices of 42, 14, 33 and 5%. Table 2 shows the immunophenotypic results. The control group of well-differentiated pancreatic NETs (*n* = 5), including a well-differentiated pancreatic NET with ectopic ACTH secretion (*n* = 1), displayed no staining with T-Pit. The original primary pituitary tumors were available for review in 2 cases and showed expression of T-Pit in each.

**Table 2:** The immunophenotype of the respective cases showing consistent expression of T-PIT and concordance between pituitary and liver samples

Case	Tissue	CGA	CDX2	TTF-1	Islet1	INSM1	OSCAR	T-PIT	Ki67 (%)
1	Liver	+	–	–	–	+	NA	+	42
2	Pituitary	+	–	–	–	NA	+	+	2
2	Liver	+	–	–	–	+	NA	+	14
3	Pituitary	+	–	–	–	+	NA	+	3
3	Liver	+	–	–	–	+	+	+	33
4	Liver	+	–	–	–	–	+	+	5

## Discussion

We present the first case series of pituitary carcinomas which metastasized to the liver and mimicked well-differentiated NETs. Histologically and cytologically, all showed characteristic features of well-differentiated NETs. Due to their rarity in comparison with other NETs, 3 of these cases presented as diagnostic dilemmas and two were initially interpreted as well-differentiated NETs of likely gastroenteropancreatic origin (Patient #1 and Patient #3). In the case of patient #1, the correct diagnosis was made upon intradepartmental consultation and consensus with the several study authors. In the case of patient #3, the presence of a concurrent pancreatic mass was confusing, but because of the history of a prior pituitary NET and history of refractory Cushing syndrome, the possibility of a metastasis from the pituitary was considered. T-Pit immunohistochemistry was positive and thus confirmed the diagnosis. Others have reported the utility of T-Pit in the evaluation of pituitary neoplasia as a specific corticotroph marker [19, 20]. The cases initially diagnosed as likely of gastrointestinal origin were recognized after referral to our practice specialty centers where the original diagnoses were questioned after a clinical work-up. The fourth case (Patient #2) was identified retrospectively in our archives. None of the cases were CDX2, TTF-1 or Islet 1 positive.

The WHO classifications of pituitary and gastroenteropancreatic NETs differ. The diagnosis of pituitary carcinoma requires recognition of metastasis, whereas for gastroenteropancreatic neuroendocrine neoplasms, the diagnosis of neuroendocrine carcinoma requires that the lesion is histologically poorly differentiated. Using the pancreas as example, since this was the presumed primary in one of our cases, poorly differentiated pancreatic neuroendocrine carcinomas may show features of small cell carcinoma or large cell neuroendocrine carcinoma [11] and differ from well-differentiated pancreatic NETs in their biology with more frequent inactivation of *SMAD4*, *RB1* and *TP53 and* no loss of function of *ATRX and DAXX* [21–23]. For visceral neuroendocrine tumors, both well-differentiated NETs and poorly differentiated neuroendocrine carcinomas may metastasize but their histologic differential diagnoses are typically different. For example, well-differentiated NETs may be mimicked by acinar cell carcinoma, low grade renal cell carcinoma, low grade adenocarcinoma, solid pseudopapillary neoplasm and glomus tumors, whereas the differential diagnostic considerations in poorly differentiated neuroendocrine carcinomas include undifferentiated carcinoma, small round blue cell sarcomas, and high-grade hematolymphoid neoplasms. The rarity of pituitary carcinoma leads it not to be considered among the much more common previously mentioned considerations, particularly as a differential for well-differentiated neuroendocrine tumors.

The value of identifying the origin for well-differentiated NET involving the liver is imperative. For the bedside physician, the site of origin provides information regarding potential surgical interventions, other potential locoregional or systemic therapies, prognostication and follow-up strategies for local disease control.

Pituitary carcinomas, defined by the presence of craniospinal and/or systemic metastases, are very rare, accounting for less than 0.5% of all pituitary tumors [5, 6, 24, 25]. A recent single institution report disclosed only 4 cases over a 15-year period including 1055 consecutive pituitary neuroendocrine neoplasms [6]. To date, there are no reliable morphologic, immunohistochemical or molecular markers of the primary tumor to confirm malignancy or metastatic potential. Whereas, some pituitary carcinomas present as aggressive tumors ab initio, most present as pituitary NET and progress with a variable number of recurrences before developing metastasis. In our series, there was a substantial lag time from the initial diagnosis of pituitary NET to developing metastases, ranging from 16 to 72 months. Given the rarity of this progressive clinical situation, the authors do not advocate for routine immunohistochemistry to exclude pituitary primaries. Rather, the authors suggest that in cases of histologically well-differentiated NETs, the clinical history should be carefully evaluated. Routine immunohistochemical markers (CDX2, SATB2, Islet 1 and TTF-1) may help identify one of the more common primary sites, but if a pituitary tumor was previously diagnosed, additional immunohistochemistry (including pituitary hormones and/or pituitary transcription factors) may be helpful to evaluate for the rare possibility of pituitary carcinoma. Similar to our findings, most pituitary carcinomas are either prolactin or ACTH-secreting [15, 26, 27]. Prolactin secreting carcinomas express transcription factors Pit-1 and ER while ACTH-secreting carcinomas express T-PIT. Pit-1 will be immunoreactive in pituitary carcinomas of the somatotroph, lactotroph, and thyrotroph lineages while SF1 and GATA3 will be immunoreactive in the carcinomas of the gonadotroph lineage. Notably, rare pancreatic neuroendocrine tumors may produce ACTH resulting in paraneoplastic Cushing syndrome, therefore the presence of Cushing syndrome does not rule out a pancreatic primary tumor [28]. Two prior case reports of corticotroph carcinoma and a single case series including one case of corticotroph carcinoma with liver metastases have been reported in the literature [29–31].

T-PIT is a transcription factor which is expressed in corticotroph and melanotroph cells exclusively [32]. T-PIT was developed as a tissue biomarker for identification of non-neoplastic and neoplastic corticotrophs [20]. Subsequently, the antibody became clinically available and is part of the panel noted in the 2017 WHO Classification of tumors of the pituitary gland for diagnosis of corticotroph adenomas [33]. Expression of T-PIT by the tumor cells in each of these cases confirmed the diagnosis and origin of the tumors involving the liver.

## Conclusion

In conclusion, we present a series of pituitary carcinomas which closely mimicked well-differentiated NETs of visceral origin. Owning to rarity of pituitary carcinoma, these cases presented diagnostic challenges. When encountering a NET with liver involvement, accurate diagnosis of the site of origin can be aided by ancillary laboratory, imaging studies and clinical context including consideration of rare primary sites such as the pituitary gland. Confirmatory immunohistochemistry can be used if a history of a pituitary tumor is noted.

## Data Availability

Can be provided upon request.

## Abbreviations

NETs: Neuroendocrine tumors
ACTH: Adrenocorticotropic hormone
GI: Gastrointestinal
PitNET: Pituitary neuroendocrine tumor
WHO: World Health Organization
CT: Computed tomography
MRI: Magnetic resonance imaging
